# X-ray interferometry without analyzer for breast CT application: a simulation study

**DOI:** 10.1117/1.JMI.7.2.023503

**Published:** 2020-03-26

**Authors:** Jingzhu Xu, Kyungmin Ham, Joyoni Dey

**Affiliations:** a Louisiana State University, Department of Physics and Astronomy, Baton Rouge, Louisiana, United States; b Louisiana State University, Center for Advanced Microstructures and Devices, Baton Rouge, Louisiana, United States

**Keywords:** analyzer-less phase-contrast breast computed tomography, modulated phase-grating interferometry, no analyzer phase-contrast x-ray, phase-contrast breast computed tomography

## Abstract

**Purpose:** We investigate an analyzer-less x-ray interferometer with a spatially modulated phase grating (MPG) that can deliver three modalities (attenuation image, phase image, and scatter images) in breast computed tomography (BCT). The system can provide three x-ray modalities while preserving the dose to the object and can achieve attenuation image sensitivity similar to that of a standard absorption-only BCT. The MPG system works with a source, a source-grating, a single phase grating, and a detector. No analyzer is necessary. Thus, there is an approximately 2x improvement in fluence at the detector for our system compared with the same source–detector distance Talbot–Lau x-ray interferometry (TLXI) because the TLXI has an analyzer after the object, which is not required for the MPG.

**Approach:** We investigate the MPG BCT system in simulations and find a clinically feasible system geometry. First, the mechanism of MPG interferometry is conceptually shown via Sommerfeld–Rayleigh diffraction integral simulations. Next, we investigate source coherence requirements, fringe visibility, and phase sensitivity dependence on different system parameters and find clinically feasible system geometry.

**Results:** The phase sensitivity of MPG interferometry is proportional to object–detector distance and inversely proportional to a period of broad fringes at the detector, which is determined by the grating spatial modulation period. In our simulations, the MPG interferometry can achieve about 27% fringe visibility with clinically realistic BCT geometry of a total source–detector distance of 950 mm and source–object distance of 500 mm.

**Conclusions:** We simulated a promising analyzer-less x-ray interferometer, with a spatially sinusoidal MPG. Our system is expected to deliver the attenuation, phase and scatter image in a single acquisition without dose or fluence detriment, compared with conventional BCT.

## 1 Introduction

According to statistics from the Centers for Disease Control and Prevention, breast cancer is the most common cancer—one in eight American women will develop it sometime during the course of her life. It is the second-highest cause of death among women in the United States. The 5-year relative survival rate of female breast cancer approaches to 90% for cases detected at an early stage, but only to about 22% for stage IV.^1^ Therefore, screening technologies that can detect breast cancer in the early stage without any signs or symptoms, are necessary for women.

Absorption x-ray mammography is the prevalent technique for breast-cancer screening for women above age 40. However, in mammography, spatial overlap of soft tissues in 2D breast projections may increase the risk of false-positive and false-negative cases in screening and diagnosis. Dedicated computed tomography (CT) to breast imaging (BCT) and tomosynthesis systems has recently been developed to represent 3D anatomic structures of uncompressed breasts to overcome limitations of tissue superposition and breast compression in mammography.^2^^–^^7^ In current clinical-based BCT prototypes at the University of California at Davis (UC Davis)^4^^–^^6^ and the Koning Corporation (University of Rochester),^7^^–^^9^ the breasts are scanned by a cone-beam x-ray in pendant geometry in which patients lie in the prostrate position on a table with an opening that allows the breasts to pass through without compression. The UC Davis prototype provided a high spatial resolution using a complementary metal-oxide semiconductor flat-panel detector, which is able to reach 50-μm pixel size.^4^^–^^6^ In January 2015, the Food and Drug Administration (FDA) approved the U Rochester BCT prototype for breast diagnostic imaging in the United States.^8^^,^^9^ Other studies have investigated the feasibility, flat-panel detectors, and spectral optimization for BCT systems.^10^^–^^12^

Phase-contrast x-ray provides not only conventional tissue attenuation provided by regular x-ray and CT but also images based on x-ray phase-shift and scatter (dark-field) within the same scan. Recently, Talbot–Lau x-ray interferometry (TLXI)^13^^–^^16^ has shown the potential to improve detection accuracy of mammography^17^^–^^19^ and microcalcification classification,^19^^,^^20^ helping detection and early diagnosis. Phase images identified trifocal tumors where x-ray absorption images failed.^17^ Complementary information from absorption and scatter images^20^ distinguished oxalate versus hydroxyapatite microcalcifications, providing a noninvasive scoring of malignancy or premalignancy risk. For soft-tissue imaging, for x-ray energy range of interest, the real part of the reflective index δ is about ∼1000 times the imaginary part β (related to the attenuation) and provides complementary information from the attenuation image. Thus, x-ray interferometry has the potential to yield higher detection sensitivity and specificity than conventional mammography^21^ or BCT.

Our eventual goal is to build a multicontrast BCT system, yielding similar quality attenuation images, while providing phase and scatter images without increasing the dose to patients. Toward this goal, we show a potential design in simulations in which we find a feasible system geometry and the grating parameters, and we address critical issues such as effects of source coherence, source-spectrum on fringe visibility, focal spot, phase sensitivity, etc.

The two interferometry methods currently at the forefront are the TLXI^13^^–^^16^ and the far-field interferometry by Miao et al.^22^^,^^23^ from the National Institutes of Health. While Talbot–Lau interferometry has made the greatest clinical stride in the mammogram domain,^19^^–^^21^^,^^24^^–^^26^ an absorption grating (analyzer) is needed to see interference patterns with standard cost-effective x-ray detectors, which is detrimental from a dose/fluence consideration. Recently, Miao et al.^22^^,^^23^ built the far-field x-ray interferometry. This eliminated the need for the analyzer as it uses two (or three) phase gratings with slight differences in pitch between them to create a low-varying “beat-frequency.” The ensuing moiré pattern fringes are directly visible with a standard detector (without the analyzer grating), reducing dose about twofold.^22^^,^^23^ One drawback is that two or three 400-nm phase gratings are required for that system^22^^,^^23^ to obtain fringe patterns. Also, the source-to-detector distance is from 1.7 to 2 m for the Miao et al. systems,^22^^,^^23^ which may be challenging to achieve clinically. The large source-to-detector distance also reduces the fluence at the detector.

Other innovative methods have required a special stepped source to eliminate the absorption grating^27^ or spectral detectors for grating-less designs.^28^^,^^29^

In conference presentations,^30^^,^^31^ we have demonstrated, in simulations, a new, simpler, clinically practical near-field system that uses a single-phase grating and no analyzer. The phase gratings investigated were spatially modulated phase gratings (MPGs) with either rectangular modulation or truncated quadratic modulations.^30^^,^^31^ The MPG system will still require a source coherence $G_{0}$ grating.

To the best of our knowledge, only our group is exploring the modulation of phase gratings for phase-contrast x-ray. Our prior conference publications on very preliminary MPG designs (rectangular and quadratic modulations) show fringe patterns for an intended mammography system. No combined analysis of source coherence and spectrum or sensitivity analysis was included. In this paper, we study a BCT of clinical dimensions with an improved sinusoidal MPG (which showed improved performance over rectangular or quadratic) and address critical issues like effects of source coherence, source spectrum on fringe visibility, focal-spot considerations, phase sensitivity, etc.

The sinusoidal was chosen as it has no sharp cutoff and is expected to yield better fringe visibility and other system characteristics. Also, it can be approximated as a triangular “modulation” of the grating. In a very different concept, for TLXI, “saw-tooth” grating elements were used instead of standard “binary” grating, improving contrast and compactness.^32^ This is physically and functionally different from our MPG case. For the MPG case, the height of the elements is slow-varying in a given functional form (such as a sinusoid), while in the TLXI work with saw-tooth grating,^32^ the grating structure does not have a height changing modulation of a base grating and requires an analyzer for imaging with standard x-ray detectors.^32^

We also note that the proposed system is physically and functionally distinct from another analyzer-less system^22^^,^^23^ by Miao et al. Our system uses a single phase grating with modulated structures (MPG) while the system^22^^,^^23^ by Miao et al. uses three gratings with standard structure (i.e., without modulation). Functionally, the MPG versus multiple standard phase gratings makes our system compact (<1  m) compared with the Miao et al. system (∼2  m), which makes our system clinically feasible.

For the sinusoidal MPG system, we show the intensity carpet for monoenergetic parallel source and realistic source coherence effects with polychromatic spectrum. We show the fringe-visibility and phase-sensitivity dependence on key grating parameters and a potential design after considering source coherence and polychromatic spectrum.

## 2 Methods

### 2.1 System Diagram and Simulation Equations

**Fig. 1:**
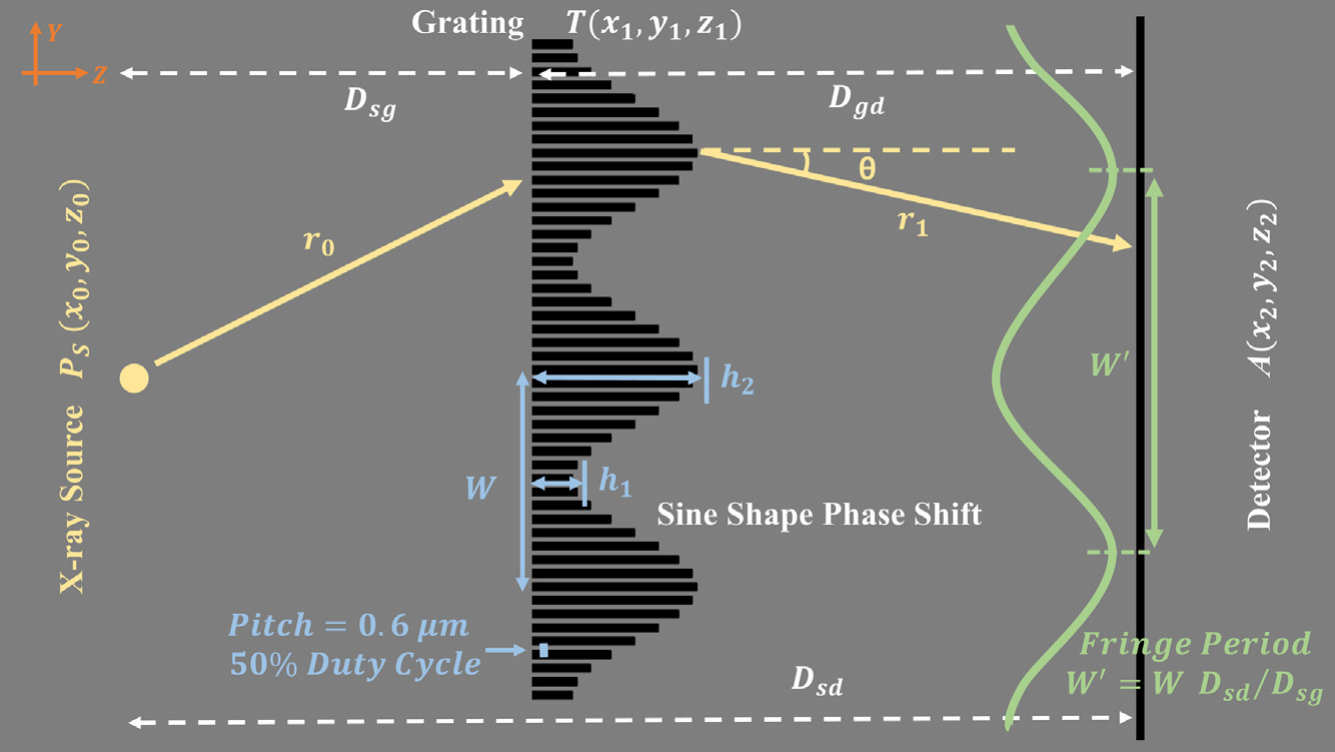
Schematic system diagram (not to scale): source, grating, and detector. The source is simplified to a point source here, but in reality, there is an x-ray tube and source-grating (with composite line sources). The grating-to-detector distance $D_{gd}$ can be 45 to 75 cm, making it a compact system with a source-to-detector distance $D_{sd}$ of ∼1  m. The grating is a special one with a sinusoidal modulation of period W over a structure of (smaller) pitch P. The heights $h_{1}$ and $h_{2}$ are the heights presented to the x-ray beam to shift the phase by certain amounts (for example, π/4 and π) for the peak-wavelength. Through geometric magnification $D_{sd}/D_{sg}$ of W, broad sinusoidal fringes with period $W^{\prime }$ are displayed on the detector.

The main idea behind the MPG system is to use a grating with a modulated slow-varying function superposed, as shown in the schematic diagram in Fig. 1. Such a function may be achieved by gradually changing the spatial height of the grating. We have a constant height grating of $h_{1}$ and a varying height grating of $h_{2}$. This will create sampling patterns or fringes with the spacing we require to image with standard CT/x-ray detector resolution.

To demonstrate the operation of such a system, we simulated a system shown in Fig. 1 using the Sommerfeld–Rayleigh diffraction integral (SRDI).^33^^,^^34^ However, before delving into the detailed design rationale behind the system, we briefly outline the simulation equations and verify with a Talbot carpet with a typical phase grating used for a Talbot–Lau system.

#### 2.1.1 Simulations

The x-ray transmission function through a phase grating in a plane perpendicular to the x-ray incidence direction on the z-axis is defined as follows:

$$T(x,y)=A(x,y)e^{j\phi (x,y)},$$ (1)

where ϕ(x,y) is the z projection phase shift determined by the grating spatial structure and A(x,y) is the corresponding amplitude transmission due to attenuation of x-rays.

The amplitude of the diffracted x-ray wave at the detector is obtained by evaluating the SRDI formula for the Huygens–Fresnel principle. In Fig. 1, $D_{sg}=z_{1}-z_{0}$ denotes the distance between the source and grating on the z-axis. $D_{gd}=z_{2}-z_{1}$ denotes the distance between the grating and detector on the z-axis. According to the SRDI,^33^ the amplitude of the x-ray on the detector plane is

$$A(x_{2},y_{2},z_{2})=\frac{1}{j\lambda }\iint U(P_{s})\cdot \frac{e^{jkr_{0}}}{r_{0}}\cdot T(x_{1},y_{1},z_{1})\cdot \frac{e^{jkr_{1}}}{r_{1}}\cdot \cos\;\theta dx_{1}\;dy_{1},$$ (2)

and the intensity is

$$I(x_{2},y_{2},z_{2})={\left|A(x_{2},y_{2},z_{2})\right|}^{2},$$ (3)

where $k=\frac{2\pi }{\lambda }$ is the wave number, λ is the wavelength, $U(P_{s})$ is the x-ray source wave function at $\left(x_{0},y_{0},z_{0}\right)$, $r_{0}$ is the distance between source and point $\left(x_{1},y,z_{1}\right)$ on the grating given by $r_{0}=\sqrt{D_{sg}^{2}+{\left(x_{1}-x_{0}\right)}^{2}+{\left(y_{1}-y_{0}\right)}^{2}}$, $r_{1}$ is the distance between the grating point $\left(x_{1},y_{1},z_{1}\right)$ to detector point $\left(x_{2},y_{2},z_{2}\right)$ given by $r_{1}=\sqrt{D_{gd}^{2}+{\left(x_{2}-x_{1}\right)}^{2}+{\left(y_{2}-y_{1}\right)}^{2}}$, and θ is the angle between $\overset{\to }{r_{1}}$ and the normal to the plane of grating. The cos θ term is also given by $\cos\;\theta =\frac{D_{gd}}{r_{1}}$.

In the initial stage of design, the source wave function $U(P_{s})$ is assumed as a parallel beam of x-rays. Therefore, Eq. (2) can be simplified as follows:

$$A(x_{2},y_{2},z_{2})\propto \;\frac{D_{gd}}{j\lambda }\iint T(x_{1},y_{1},z_{1})\cdot \frac{e^{jkr_{1}}}{r_{1}^{2}}dx_{1}\;dy_{1},$$ (4)

where we consider a planar wave incident on grating. Since the distance of the plane wave to grating is assumed to be infinity, we can ignore that term. We replaced the cos θ (between the $r_{1}$ and z axis) with $\frac{D_{gd}}{r_{1}}$, resulting in a $r_{1}^{2}$ term appearing in the integral in Eq. (4). When displaying the intensity carpet, we show the normalized intensities at each $D_{gd}$ to better visualize the fringe visibility.

#### 2.1.2 Talbot carpet

**Fig. 2:**
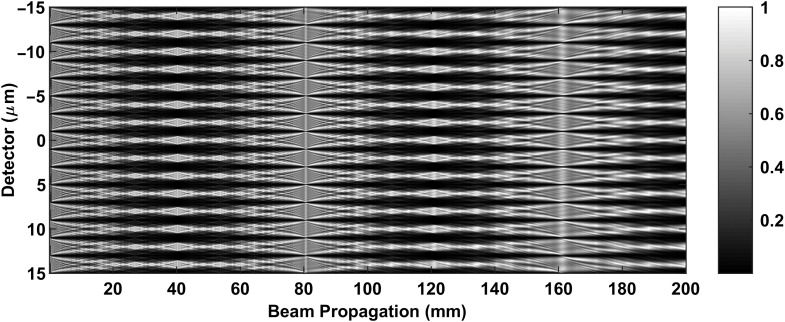
Normalized Talbot carpet for a 4-μm π-phase grating typically used in a Talbot–Lau interferometer.^15^^,^^16^ The first and third order Talbot distances from the grating are 40.3 and 120.9 mm, respectively.

First, to verify our computation, we simulate the Talbot carpet (Fig. 2) for the Talbot–Lau system for a 4-μm standard $G_{1}$ phase grating that is typically used in the TLXI system.^15^^,^^16^ We cover distances of 0 to 200 mm from grating. The pattern is as expected for the π-shift grating, and we observe the first and third Talbot distances at correct distances of 40.3 and 120.9 mm, respectively.^16^ We note that the fringe pattern periodicity is only about 4  μm; hence, an analyzer (absorption grating) is required to observe patterns on a typical clinical x-ray detector (35 to 75  μm pixel size).

### 2.2 Phase Grating Design

In what follows, we explain our grating design step-by-step using the above simulator.

**Fig. 3:**
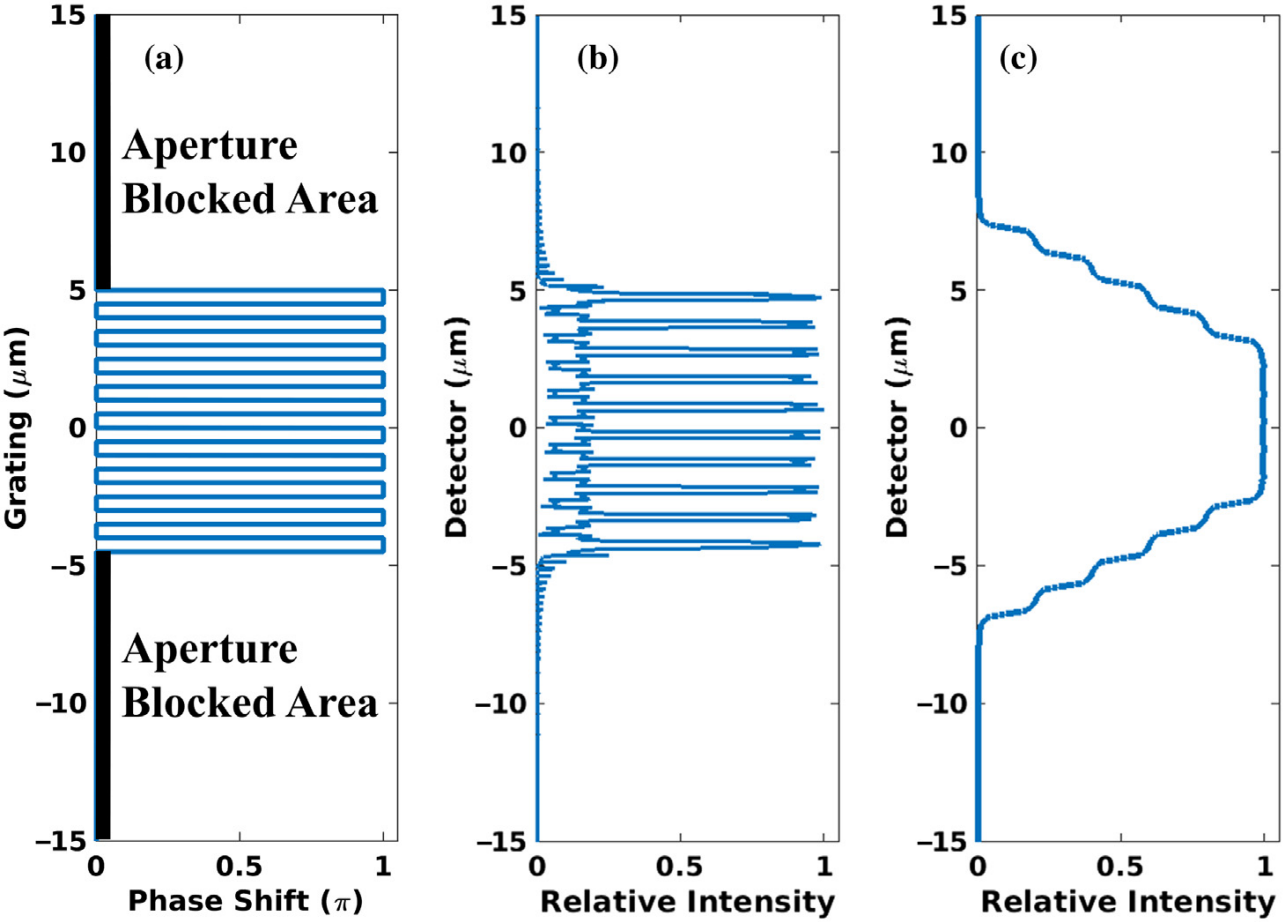
(a) Spatial structure of a phase grating with aperture. Pitch width is 1  μm and aperture open width is 10  μm. (b) Normalized intensity with high resolution (0.1-μm pixel size) at the first-order Talbot distance 2.5 mm downstream of the grating with 25 keV x-ray. (c) Normalized intensity after convolving with a 5-μm width window function.

As shown in Fig. 2, without the analyzer, the intervals of intensity fringes are much smaller than the pixel size of a typical high-resolution flat-panel detector (50  μm), making the fringe pattern impossible to be distinguished. As a result, there will only be a bright spot displayed on the detector. Figure 3 shows this phenomenon in simulations: Fig. 3(a) is the 1-μm pitch grating spatial structure with a 10-μm aperture, Fig. 3(b) is the intensity pattern in high resolution, and Fig. 3(c) is the intensity smoothed with a 5-μm box filter. The plot is shown in the original resolution for ease of comparison.

**Fig. 4:**
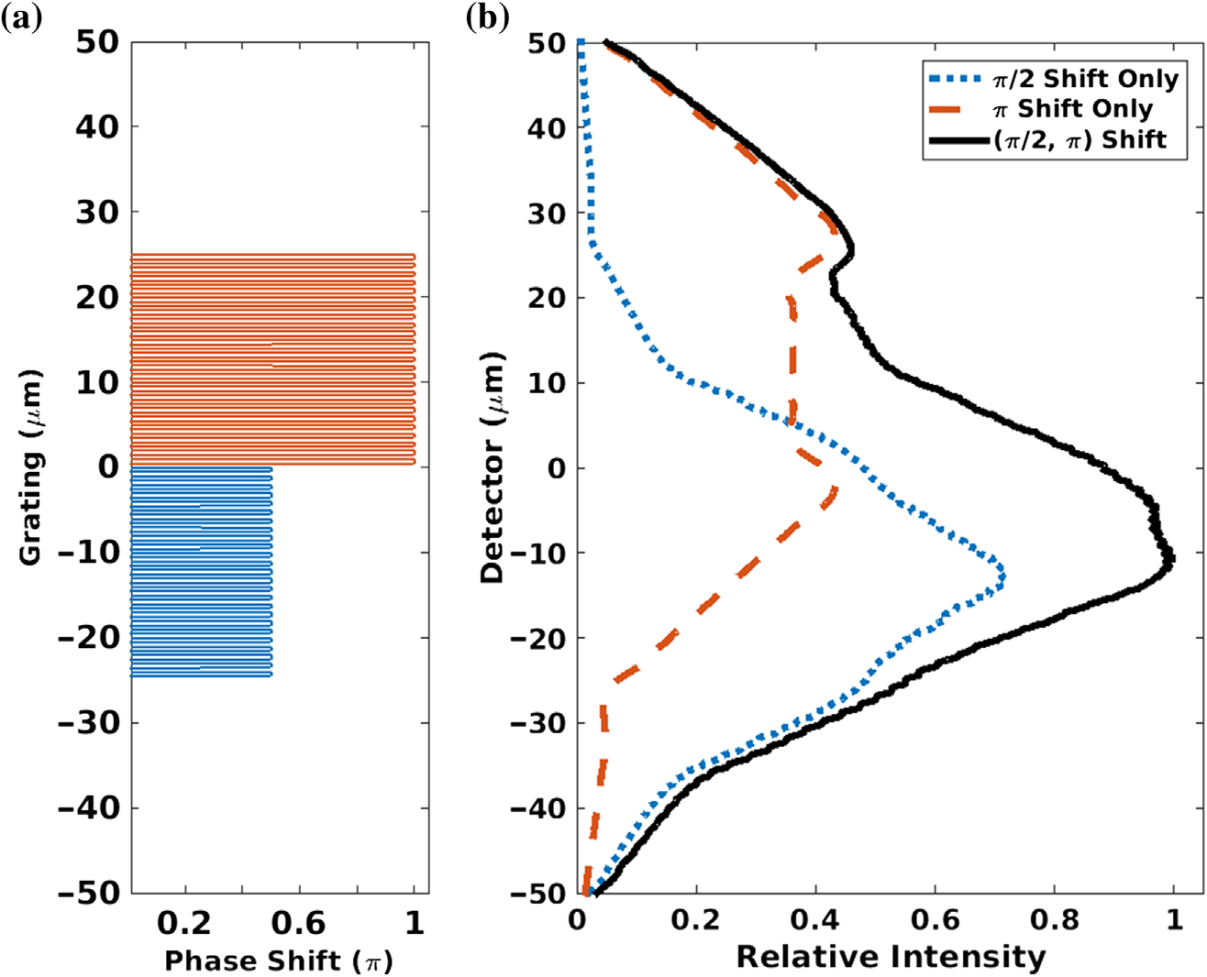
(a) Spatial structure of a grating combined by two linear gratings with π/2 and π phase shifts with 1-μm pitch. (b) Normalized intensities at 30 cm downstream of the grating with 25 keV x-ray. The red and blue dashed lines show the intensity of response due to the individual gratings (π shifter in blue and π/2 shifter in red).

**Fig. 5:**
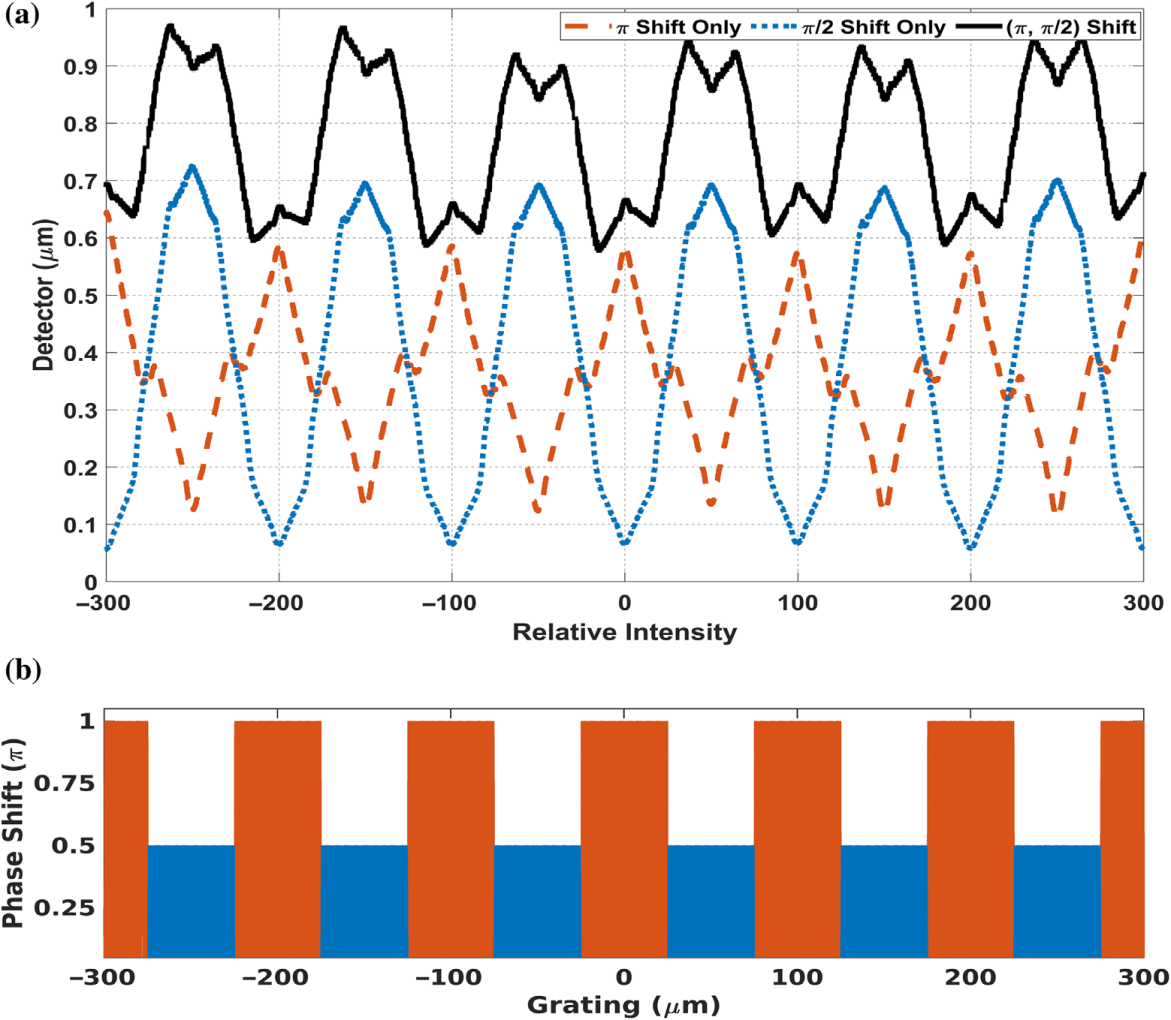
(a) The entire fringe pattern (black curve) due to repeated units of low–high linear phase-shift grating combination. The red and blue curves indicate individual wavefront intensities modulated by the grating units at π/2 and π, respectively, shown in (b). (b) The entire grating is plotted to show corresponding alignment of grating units. Here, the pitch period is 1  μm, x-ray energy is 25 keV, grating-to-detector distance is 300 mm, and the high–low pattern is repeated at 100  μm (W=100  μm).

The amount of phase-shift modulation from grating is determined by the phase-shift or the height of the grating. A grating with a different phase shift produces spots with different brightness for the same grating-to-detector distance. To demonstrate this point, two gratings (inside the same structure) with different heights (phase shifts) are placed adjacently as in Fig. 4(a); then, intensities of the two individual gratings and the intensity of the combined grating are shown in Fig. 4(b). Broad fringes composed of different bright spots distributed at regular intervals can then be created by repeating the lower- and higher-level combined phase grating units on the plane perpendicular to the optical axis. Figure 5 shows an entire fringe pattern created by this method. The period of broad fringe pattern can be controlled by the width of multiple slits. A spot with high brightness is the crest of a fringe and adjacent spots with lower brightness are the trough. In Figs. 4 and 5, the x-ray energy is 25 keV, and the grating-to-detector distance is 300 mm. The grating function is sampled at 1 nm, and the detector is sampled at 10 nm.

To demonstrate the concept of the phase grating design, a rectangular-pattern grating was useful (Figs. 3–5), but better fringe visibility is obtained from a sinusoidal pattern (such as shown in Fig. 1), where the superposing structure is sinusoidal. In what follows, we assume a sinusoidal pattern to compute our carpets and visibility.

### 2.3 Performance Simulations of Our System

We performed a series of simulations for our system with increasing complexity, beginning with (1) the intensity carpet and fringe visibility analysis along the z direction with a parallel source at 40 keV. The MPG-to-detector distances are varied as z=450 to 750 mm. We also analyzed the dependence of fringe visibility on energy, pitch, and grating spatial modulation period W, at different MPG-to-detector distances. (2) The intensity carpet with a point source at 40 keV and 250 mm source-to-MPG distance is shown. Degradation of visibility due to polychromatic point source with a typical spectrum for BCT is shown. (3) We compute the spatial coherence requirement and show the fringes and fringe visibility for a line source compared with a point source. Each of these is explained in more detail below.

#### 2.3.1 Intensity carpet and fringe analysis with mono-energetic, parallel source

A carpet for Talbot–Lau phase grating such as one shown in Fig. 2 captures the intensity of the interference pattern at different distances from the grating and is useful for determining optimal detector placement. We considered the spatial MPG (Fig. 1) with phase heights $\left(h_{1},h_{2})=(\pi /4,\pi \right)$, 0.6 to 1.2  μm pitch, and 100 to 200  μm W. For parallel source, the source-to-grating distance is assumed as infinite; thus, there is no magnification from geometry or the fringe period $W^{\prime }=W$.

We computed the fringe-intensity carpet for our grating pattern with 100  μm W at 450 to 750 mm MPG-to-detector distance. A monoenergetic (40 keV) parallel source is assumed. The grating is sampled at 1 nm, and the detectors (at each z) are sampled at 10 nm. We consider detector placement in the beam propagation direction z with 5-mm intervals. We show the carpet with two cases of pitch: 1 and 0.6  μm. And the visibility around the central axis is also calculated by $\frac{I_{\text{peak}}-I_{\text{trough}}}{\left(I_{\text{peak}}+I_{\text{trough}}\right)}\times 100\%$ at every placement and plotted with respect to z, where the $I_{\text{peak}}$ and $I_{\text{trough}}$ are the intensities at the peak and trough of the fringes, respectively.

For the same geometry, the fringe visibilities at 450 to 750 mm MPG-to-detector distances are computed and plotted for different energies between 20 and 50 keV, different grating pitches between 0.6 and 1.2  μm, and different fringe periods between 100 and 200  μm. These analyses allow for other system design considerations (low energy mammogram to higher energy CT).

#### 2.3.2 Fringe analysis with a monochromatic and polychromatic (with BCT spectrum) point source

Point-source carpet, monoenergetic: We simulated the intensity with a monochromatic (40 keV) point source for a source-to-MPG distance of 250 mm and MPG-to-detector distance varying from 450 to 750 mm. For a spherical wave from the point source in the cone-beam geometry, the fringe period $W^{\prime }$ on the detector is the grating spatial modulation parameter W scaled by the magnification factor $D_{\mathrm{sd}}/D_{\mathrm{sg}}$ (see Fig. 1).

Temporal coherence: To demonstrate that MPG can work well with a broad energy band like TLXI and propagation-based interferometers,^15^^,^^16^ we simulated the fringe intensities with a point source in the range of 26 to 60 keV individually for a given MPG with fixed phase heights $\left(h_{1},h_{2})=(\pi /4,\pi \right)$ at 40 keV. With the geometry of a source-to-MPG distance $D_{\mathrm{sg}}=250\;\mathrm{mm}$ and an MPG-to-detector distance of $D_{\mathrm{gd}}=600\;\mathrm{mm}$, the visibility of 150-μm periodic fringes for each energy was computed and is shown in Sec. 3.

Point-source, polyenergetic (BCT spectra): For a given MPG-to-detector distance of 600 mm and source-to-MPG 250 mm, we compare the fringes at the detector from a monochromatic source versus a polychromatic spectrum suitable for BCT. For example, for the UC Davis prototypes of BCT, the spectrum is set up by 60 kV with 0.2-mm Cu filter.^5^^,^^6^ The x-ray source is an aggregate of sources with different energies, each weighted by the number of photons at the typical energy in the spectrum. The sources of different energies are incoherent and responses to all of the weighted sources are added in intensity.^16^^,^^35^

#### 2.3.3 Spatial coherence requirement and source grating

We now consider two issues: one is the spatial coherence requirement of source for interference fringes to occur for our system and the other is the effect of the focal spot blur. These concepts are similar to TLXI and have been analyzed in depth for TLXI;^12^^,^^13^ we go over them briefly for our system. X-ray tubes typically used for clinical BCT or mammogram are incoherent sources. We, therefore, require a source grating $G_{0}$ such as is required of the TLXI system. $G_{0}$ is placed close to the x-ray tube focal spot. It can be thought of as a series of line sources of length $L_{s}$, which are mutually incoherent.^15^^,^^16^

For a single line source of size $L_{s}$, the corresponding projected source size $L_{s}\frac{D_{gd}}{D_{sg}}$ on the detector will be convolved with the fringe intensity profile from a point source. Since the broad fringe (>50  μm) created by MPG is composited by superfine fringes (in the order of grating pitch) with sinusoidal intensity modulation of $W^{\prime }$ (>50  μm), the degradation of visibility (due to the convolution of a line source projection, assumed as a window function, with the finite size that is smaller than one-pixel size of 50  μm) will be low.

For our geometry, when $L_{s}\frac{D_{gd}}{D_{sg}}\leq 50\;\mu m$, the line source size $L_{s}\leq 16.6\;\mu m$, where $D_{sg}$ is the distance from source grating $G_{0}$ to MPG and $D_{gd}$ is the distance from MPG to the detector. However, the coherence length for the peak wavelength is given by $l_{c}=\frac{\lambda D_{sg}}{P_{0}{ϒ}_{0}}=\frac{\lambda D_{sg}}{L_{s}}$, where λ is the peak wavelength and $P_{0}$ and ${ϒ}_{0}$ are the pitch and the open-ratio of $G_{0}$, respectively. The line-source size is the extent of the opening in one pitch period $P_{0}$ or $L_{s}=P_{0}{ϒ}_{0}$. The source coherence length $l_{c}$ has to be larger or equal to $P_{\mathrm{MPG}}$, the pitch of the MPG. With this constraint, for $P_{\mathrm{MPG}}=0.6\;\mu m$, $D_{sg}=250\;\mathrm{mm}$, and 40 keV, we would obtain $L_{s}=12.9\;\mu m$. Since fringe patterns created by different line sources should be superposed on the detector, the pitch of the $G_{0}$ grating can be calculated as $P_{0}=\frac{W^{\prime }}{D_{gd}}D_{sg}=62.5\;\mu m$, where MPG-to-detector distance $D_{gd}$ is 600 mm and the fringe period in the detector $W^{\prime }$ is 150  μm in our case. This would lead to an open-ratio ${ϒ}_{0}=\frac{12.9}{62.5}=20.64\%$. This is similar to that used in TLXI systems.^15^^,^^16^^,^^26^

For MPG with larger pitches, all else being equal, the required coherence length $l_{c}$ will be larger, line source $L_{s}$ has to be smaller, and the open ratio of $G_{0}$ has to be lowered. For 1-μm pitch MPG, $L_{s}$ has to be reduced to 7.7  μm and the open-ratio ${ϒ}_{0}$ decreases to 12.4%. These parameters are summarized in Table 1.

**Table 1:** Summarized parameters designed for spatial coherence requirements.

Geometry	Design energy	Fringe period in detector	MPG (μm)	Source grating $G_{0}$	
				Pitch $P_{0}$	Open-ratio ${ϒ}_{0}$ (%)
$D_{\mathrm{sg}}=250\;\mathrm{mm}$	40 keV	$W^{\prime }=150\;\mu m$	0.6	62.5	20.64
$D_{\mathrm{gd}}=600\;\mathrm{mm}$			1	62.5	12.4

We computed (via SRDI) the fringe pattern with a line source for the 0.6-μm $P_{\mathrm{MPG}}$ case with $L_{s}=12.9\;\mu m$, as shown in Sec. 3.

Since the source grating $G_{0}$ decouples the focal spot size of the x-ray tube and requirements of spatial coherence, the resolution of fringes is independent of the imaging spatial resolution.^15^^,^^16^ The spatial resolution or the focal spot blur for the scanning object is still determined by the focal spot size of the x-ray tube and the geometry of source, object, and detector, as in noninterferometric BCT.

### 2.4 Phase Sensitivity and Example in BCT Geometry

#### 2.4.1 Phase sensitivity

**Fig. 6:**
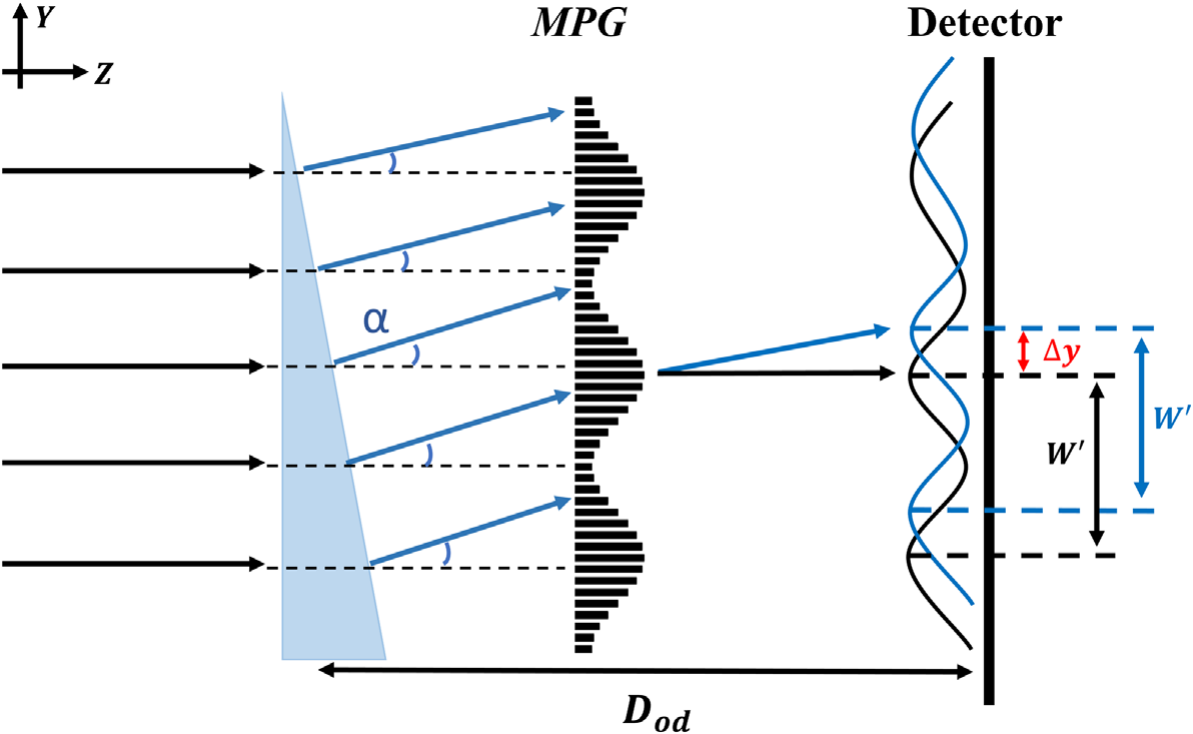
Schematic diagram of phase sensitivity in parallel beam. The refractive angle α from an object in a ramp shape with a constant differential phase shift in the y direction causes the intensity fringes shift Δy in the detector. $D_{\mathrm{od}}$ is the distance between the object and detector. The intensity fringes are in period $W^{\prime }$.

The phase sensitivity for an interferometer is defined as $S=\frac{\Delta \varphi }{2\pi \alpha }$, where α is the refractive angle caused from the object’s differential phase shift profile and Δφ/2π is the measured phase shift in intensity fringes, which is normalized by 2π.^36^ As the parallel beam shown in Fig. 6, the refractive angle α from an object’s differential phase shift profile in the y direction $\frac{\partial \Phi }{\partial y}$ is $\alpha =\frac{\lambda }{2\pi }\frac{\partial \Phi }{\partial y}$, where λ is the wavelength, Φ is the phase-shift profile of the object, and the integrated phase shift measurement $\varphi =S\lambda \int (\frac{\partial \Phi }{\partial y})dy$. The fringe intensity shifts Δy in the detector caused by refractive angle α is $\Delta y=D_{\mathrm{od}}\;\tan\;\alpha \approx \alpha D_{\mathrm{od}}$, where $D_{\mathrm{od}}$ is the distance between the object and detector. Since the normalized intensity fringe phase shift Δφ/2π can be obtained by $\Delta y/W^{\prime }$, the phase sensitivity of our MPG interferometer is calculated as $S=\frac{D_{\mathrm{od}}}{W^{\prime }}$. For the cone-beam “inverse” geometry with MPG upstream of the object $\left(D_{\mathrm{od}}<D_{gd}\right)$, following Tilman et al.,^36^ the fringe intensity shift Δy in the detector remains $\alpha D_{\mathrm{od}}$. Therefore, the phase sensitivity remains the same in the inverse geometry mode with a point source. To demonstrate that the phase sensitivity of MPG is proportional to the object-to-detector distance and inversely proportional to the intensity fringe period, we compute the fringe patterns (via SRDI) for a ramp-shaped object in a constant differential phase shift, 2π rad/mm, with different $D_{\mathrm{od}}$ (40 to 60 cm) and $W^{\prime }$ (100 to 200  μm) at 40 keV in cone-beam geometry. Then, the integrated phase-shift measurements φ are retrieved by the single-shot method^22^ without scaling by phase sensitivity. For a fixed constant $\frac{\partial \Phi }{\partial y}$, φ is proportional to phase sensitivity S for different cases of $D_{\mathrm{od}}$ and $W^{\prime }$. The variation of sensitivity versus $D_{\mathrm{od}}$ and $W^{\prime }$ is shown in Sec. 3.

#### 2.4.2 MPG example in BCT geometry

**Fig. 7:**
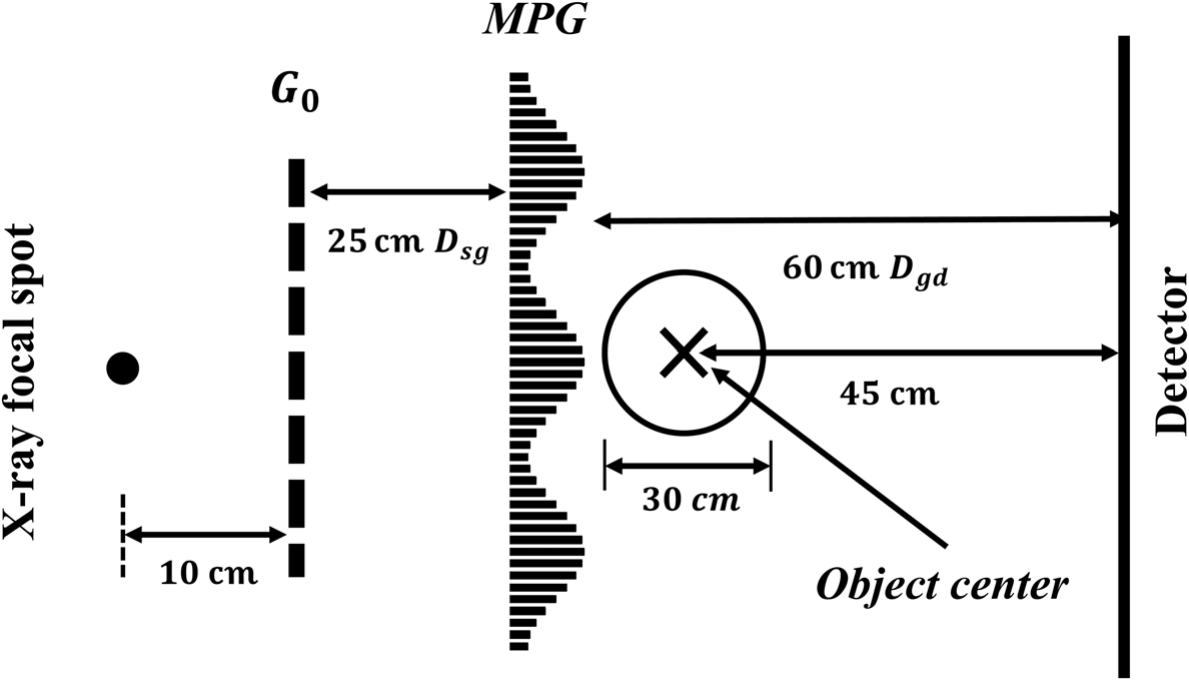
An example of MPG application in BCT geometry. The design energy is 40 keV and MPG parameters are 0.6-μm pitch and 45  μm W. The source grating $G_{0}$ with 62.5  μm $P_{0}$ and 20% open ratio is placed 10 cm away from focal spot. $D_{sg}$, $D_{gd}$, and $D_{\mathrm{od}}$ are 25, 60, and 45 cm, respectively.

According to current clinical-based BCT prototypes,^4^^–^^9^ the space between the x-ray source and the isocenter is generally ∼50 to 65 cm and the magnification factors in the range of 1.4 to 2. The circular opening on the table for hanging uncompressed breast is ∼30  cm diameter.^5^^,^^7^ The x-ray tube works in 50 to 80 kV with an Al filter or 0.2- to 0.3-mm Cu filter (UC Davis).^4^^–^^7^ We refer to these parameters to build up a similar geometry for MPG application in BCT (Fig. 7).

In our geometry, the distance from the x-ray focal spot to the isocenter (the center of object) is kept at 50 cm (to be at par with the UC Davis system). Considering there is a small space between the focal spot and x-ray tube window and installation of filter, the source grating $G_{0}$ is placed 10 cm away from the focal spot. The $D_{sg}$ and $D_{gd}$ are kept at 25 and 60 cm. Consequently, for the 0.6-μm pitch MPG, to obtain 150-μm intensity fringe period at designed energy 40 keV, the setup of the source grating (pitch $P_{0}$ and open-ratio ${ϒ}_{0}$) is the same as previous simulations in part C. The distance from the isocenter to the detector, $D_{\mathrm{od}}$, is 45 cm and the magnification factor for the object is 1.9. Therefore, the phase sensitivity $S=\frac{D_{\mathrm{od}}}{W^{\prime }}=\frac{45\;\mathrm{cm}}{150\;\mu m}=3\times 10^{3}$.

## 3 Results

### 3.1 Performance Analysis Results

#### 3.1.1 Parallel source, intensity carpet, and fringe analysis

**Fig. 8:**
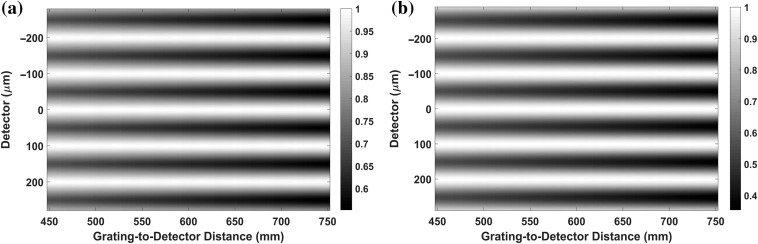
Fringe intensity carpet for parallel beam at 40 keV with 100  μm W from 45 to 75 cm MPG-to-detector distance. (a) 1-μm MPG pitch and (b) 0.6-μm MPG pitch.

The intensity carpet is shown in Fig. 8 for 1 and 0.6  μm pitch of the MPG, respectively, for W=100  μm. The fringe peak-to-peak follows the peak-to-peak of the slow-varying modulation in the grating (that is, fringe period $W^{\prime }=100\;\mu m$ here). This is as expected from the source being parallel and the concepts outlined in Figs. 4 and 5.

**Fig. 9:**
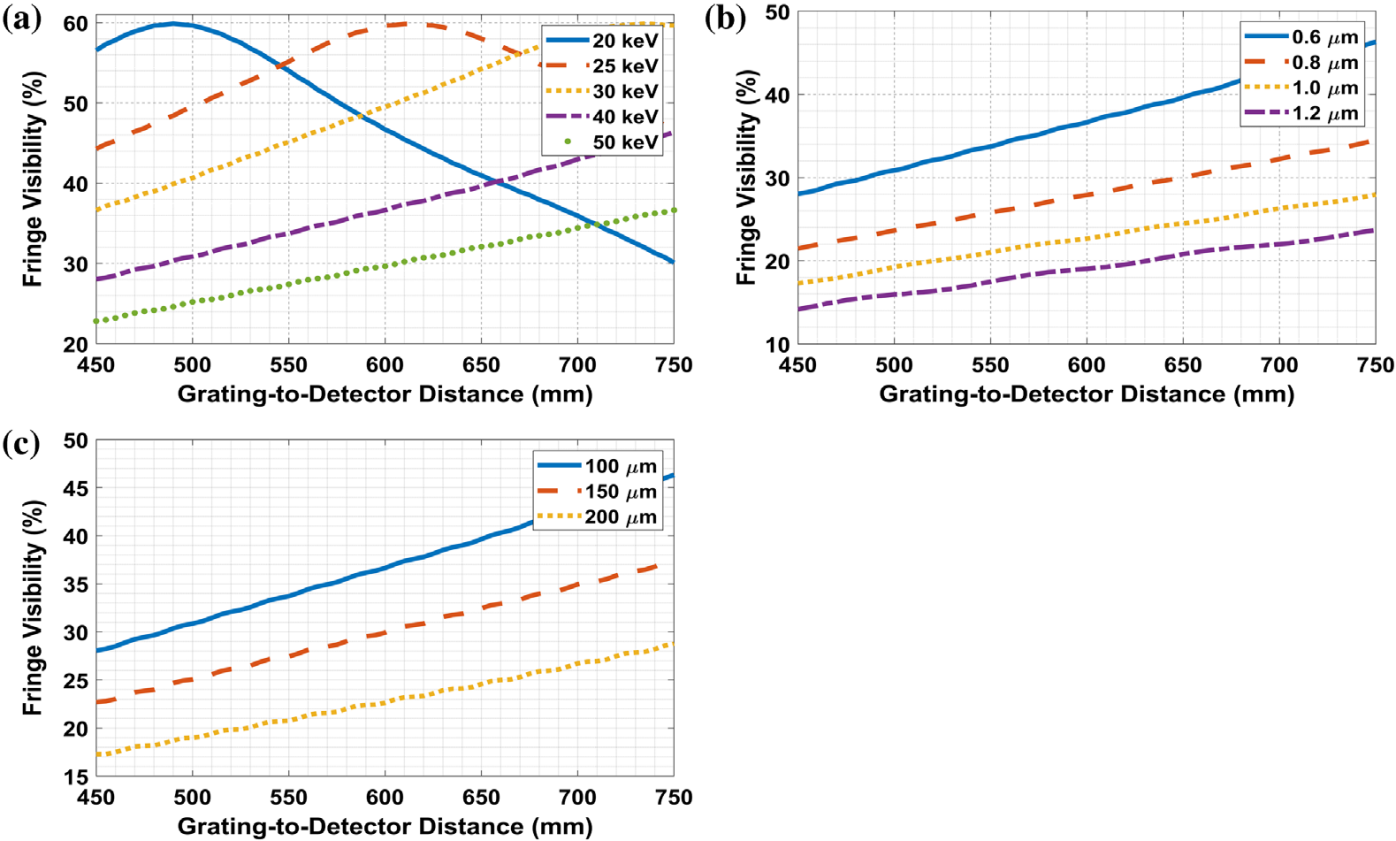
The fringe visibility analysis in parallel beam with 45 to 75 cm MPG-to-detector distance. (a) The fringe visibility with distance for different energies (in parallel beam) with 0.6-μm pitch, 100-μm W. (b) The pitch dependence of the fringe visibility at 40 keV, 100  μm W. (c) The W dependence of fringe visibility at 40 keV 0.6-μm pitch.

Figure 9(a) shows that the fringe visibilities, in general, increase with distance and decrease after reaching the maximum value. The lower energy, the shorter grating-to-detector distance needed to reach the maximum visibility. Figures 9(b) and 9(c) show the fringe visibility dependence with MPG parameters modulation period W and pitch P, showing an inverse relation with both: that is, the lower the W or P is, the higher the visibility is.

#### 3.1.2 Point source, monoenergetic intensity carpet, and fringe analysis in energy spectrum

**Fig. 10:**
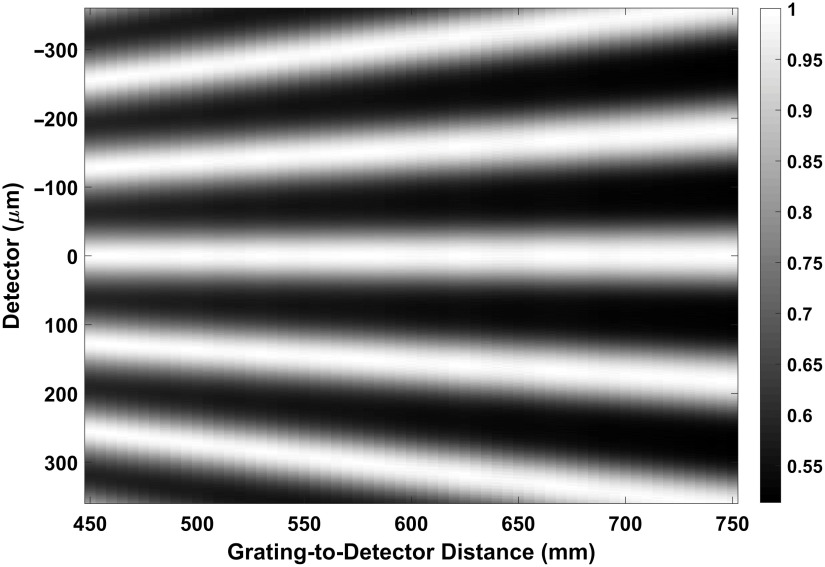
Fringe intensity carpet for a point source at 40 keV with 0.6-μm pitch in 25 cm source-to-grating distance and 45 to 75 cm MPG-to-detector distance. The fringe period is 150  μm at 60 cm MPG-to-detector distance.

The fringe intensity carpet is regenerated for a 40-keV point source, 25 cm from the MPG (0.6  μm P, 45  μm W). The carpet for 45 to 75 cm MPG-to-detector $D_{gd}$ distance is shown in Fig. 10. The fringe period $W^{\prime }$ is scaled by the geometric magnification factor, given by the ratio of $\frac{Dsd}{Dsg}$. For the detector intensity at $D_{gd}=60\;\mathrm{cm}$, the fringe period at the detector is $W^{\prime }=150\;\mu m$.

**Fig. 11:**
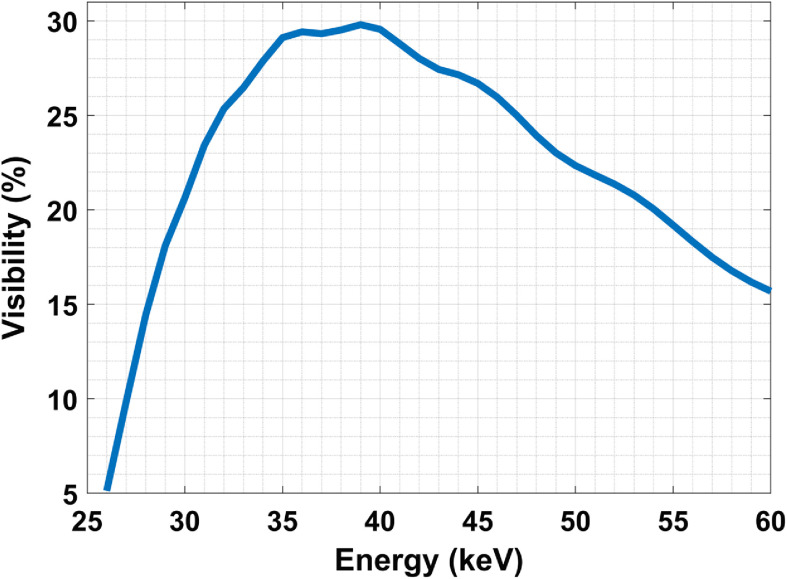
Fringe visibility dependence on energy for a point source from 26 to 60 keV with 0.6  μm pitch, in 25 cm source-to-MPG distance and 60 cm MPG-to-detector distance. The MPG phase shift is fixed at (π/4,π) for 40 keV.

Figure 11 shows the visibility dependence on energy (without spectrum amplitude variation) for a point source, 25-cm source-to-MPG distance, and 60-cm MPG-to-detector distance. The MPG phase shift is fixed at (π/4,  π) for the design energy 40 keV. As a result, the variation of 150-μm periodic fringe visibility is small (within 5%) in 40±6  keV.

**Fig. 12:**
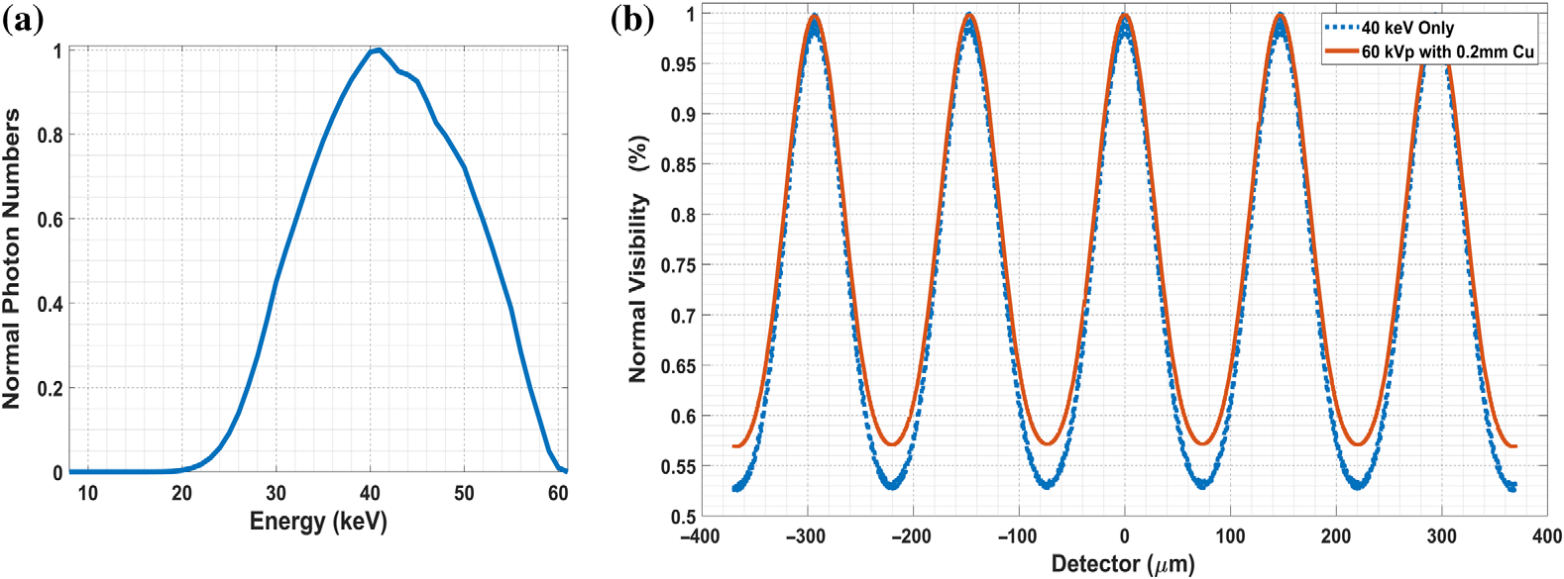
Fringe analysis in spectrum (using SRDI): (a) An example spectrum chosen for BCT with 60 kV and 0.2 mm Cu. It is generated by the online tool “simulation of x-ray spectra” from Siemens Healthcare. The mean energy mode is around 41 keV. (b) The fringe visibility (normalized) with spectrum and at monoenergetic 40 keV. The visibility dropped from 30.5% to ∼27%.

In Fig. 12(a), we chose a realistic BCT spectrum to investigate the degradation of fringes due to the polychromatic nature of the x-ray tube beam. The spectrum is derived using 60 kV, 0.2-mm Cu filter, as used in UC Davis prototype for BCT.^4^^–^^6^ Figure 12(b) shows the fringe pattern at pure 40 keV and with this polychromatic spectrum. We note that the fringe visibility dropped from 30.5% to about 27%, not a significant drop in this energy range.

#### 3.1.3 Line source

**Fig. 13:**
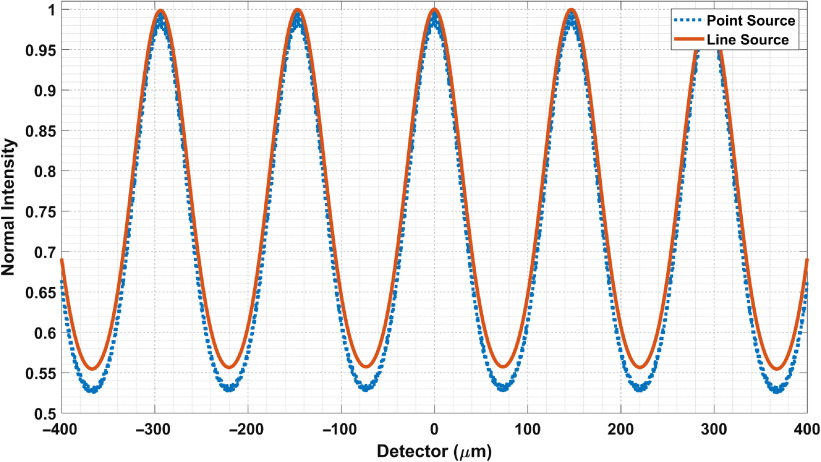
Comparison of point source and line source (using SRDI). The sources are 25 cm from MPG. The MPG pitch is 0.6  μm and $W^{\prime }=150\;\mu m$. The detector is 60 cm from MPG. We note a drop of fringe visibility from 30.5% to 28.4%.

In Fig. 13, we generated the fringes for a line source 12.9  μm, as calculated in Sec. 2. This represents an opening in $G_{0}$. The source-to-MPG distance is 25 cm, and the MPG-to-detector distance is 60 cm. The MPG parameters are 0.6-μm pitch and $W^{\prime }=150\;\mu m$. The fringe visibility drops slightly from 30.5% to 28.4%.

**Fig. 14:**
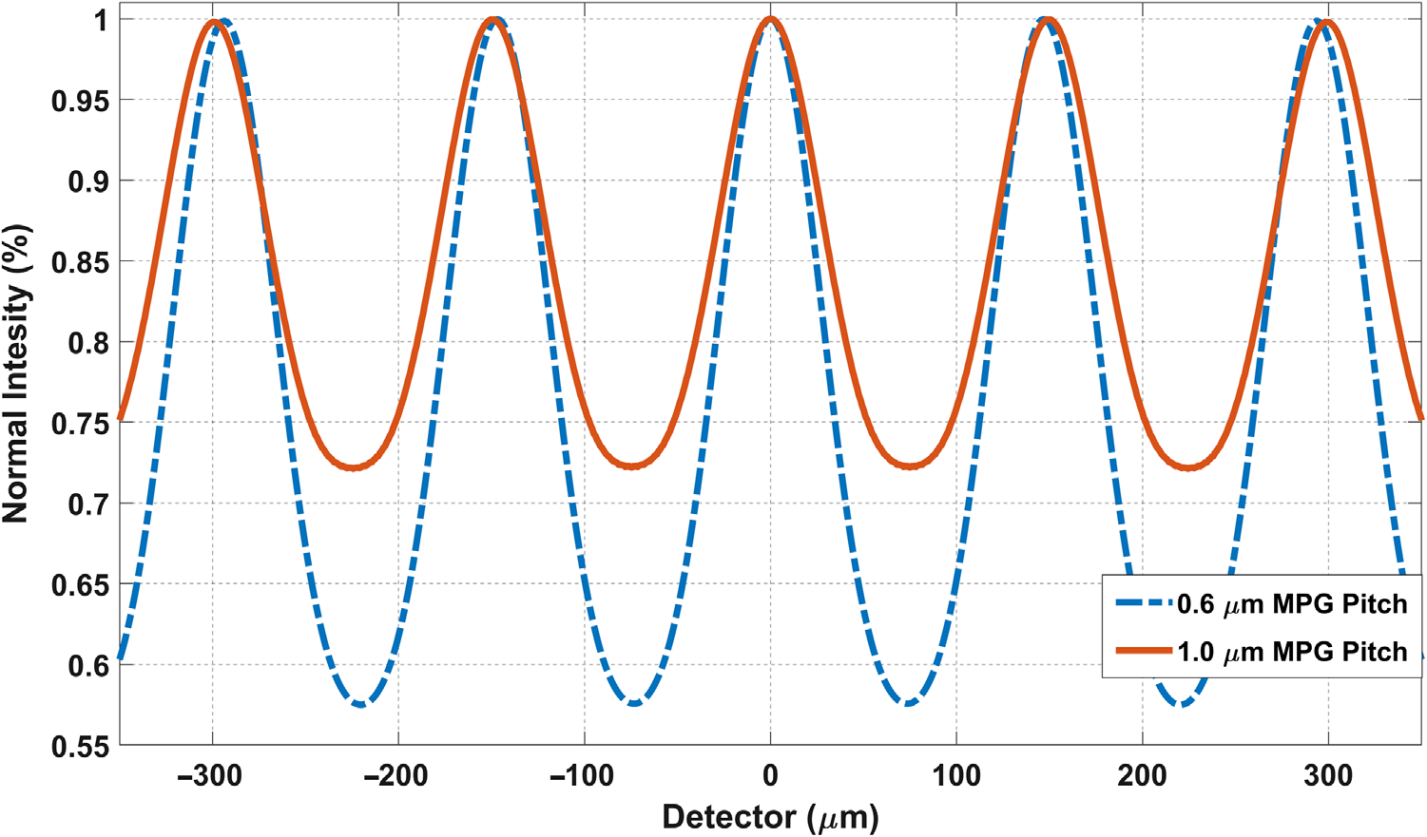
Comparison of MPG pitch (using SRDI) with spectrum and line source. The sources are 25 cm from MPG and $W^{\prime }=150\;\mu m$. The detector is 60 cm from MPG. We note a drop of fringe visibility from 27% to 16.1%.

#### 3.1.4 MPG pitch

With the same geometry, 25 cm source-to-MPG distance and 60 cm MPG-to-detector distance and the same fringe period $W^{\prime }=150\;\mu m$, we generated the fringes for 0.6 and 1  μm MPG pitch with the corresponding coherent line source size of 12.9 and 7.7  μm in a BCT spectrum [Fig. 12(a)]. The fringe visibility drops from 27% to 16.1% (Fig. 14). The results are summarized in Table 2.

**Table 2:** Summarized fringe visibilities for different simulations.

Source	Fringe period in detector	MPG pitch (μm)	Energy	Visibility (%)
Point source	$W^{\prime }=150\;\mu m$	0.6	40 keV	30.5
			Spectrum with 60 kVp and 0.2 mm Cu	27
Line source			40 keV	28.4
			Spectrum with 60 kVp and 0.2 mm Cu	27
		1.0		16.1

### 3.2 Phase Sensitivity Results

**Fig. 15:**
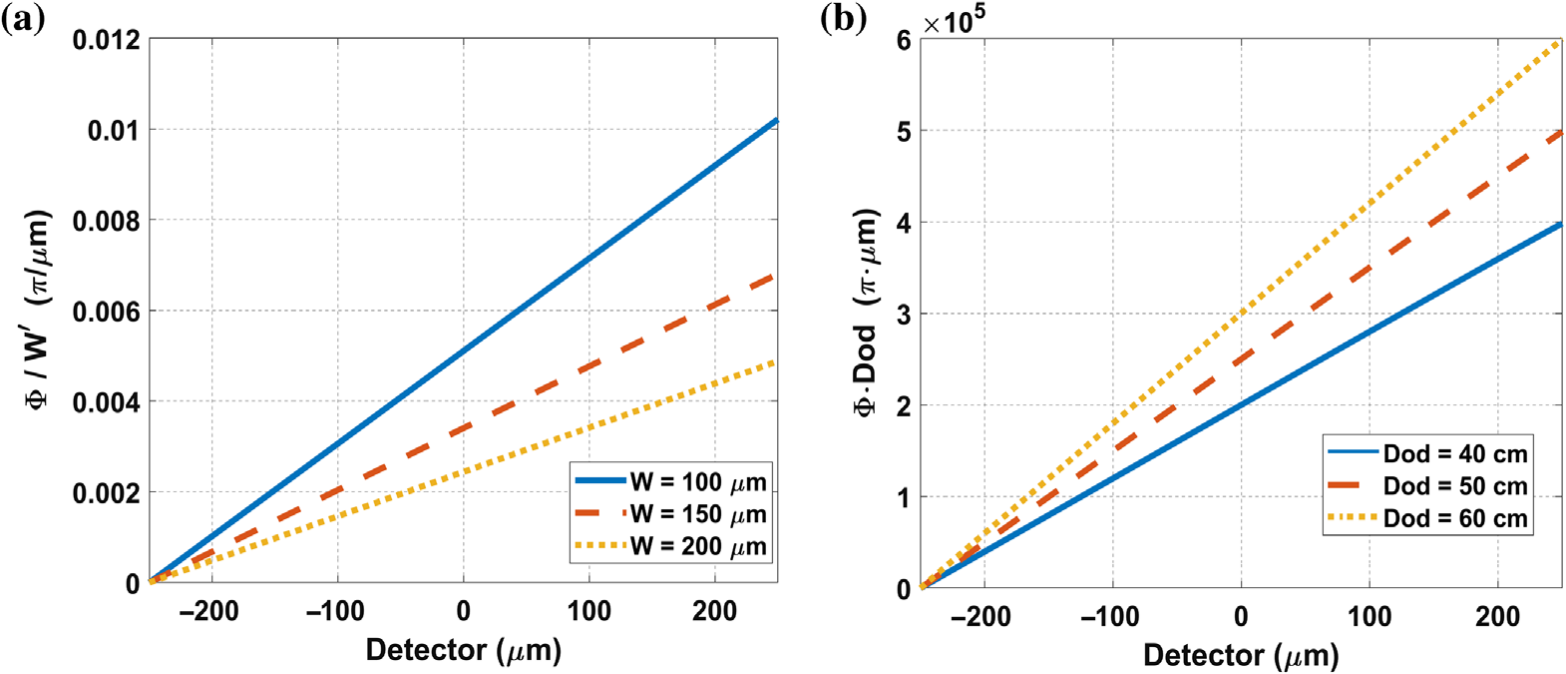
Phase-sensitivity dependence on $W^{\prime }$ and $D_{\mathrm{od}}$ (object distance). Measured with a constant differential phase shift object and integrated, obtaining a ramp across the detector with 0 to π shift in 500  μm. (a) Phase sensitivity in units of π for different $W^{\prime }$. The higher the $W^{\prime }$ is, the less the sensitivity is. (b) Phase sensitivity in units of π for different object distance. The higher the object distance is, the better the phase sensitivity is.

Figure 15 shows that the measurement phase profile φ is proportional to phase sensitivity $S=\frac{D_{\mathrm{od}}}{W^{\prime }}$ for a ramp-shaped object with $\frac{\partial \Phi }{\partial y}=2\pi \;\mathrm{rads}/\mathrm{mm}$. In Fig. 15, the measurement phase profile φ spans 500  μm in the y direction; thus, the object phase profile Φ is a ramp from 0 to π. The MPG is in 0.6-μm pitch and the source-to-MPG distance is 25 cm. In Fig. 15(a), we retrieved φ from fringe intensities with different fringe period $W^{\prime }$ at the same object-to-detector distance $D_{\mathrm{od}}=60\;\mathrm{cm}$. The scaled measurement phase profile $\frac{\varphi }{\lambda D_{\mathrm{od}}}=\frac{\Phi }{W^{\prime }}$ is inversely proportional to $W^{\prime }$. In Fig. 15(b), we retrieved φ from fringe intensities at different object-to-detector distance $D_{\mathrm{od}}$ with the same fringe period $W^{\prime }=150\;\mu m$. The measurement phase profile φ is proportional to $D_{\mathrm{od}}$.

**Fig. 16:**
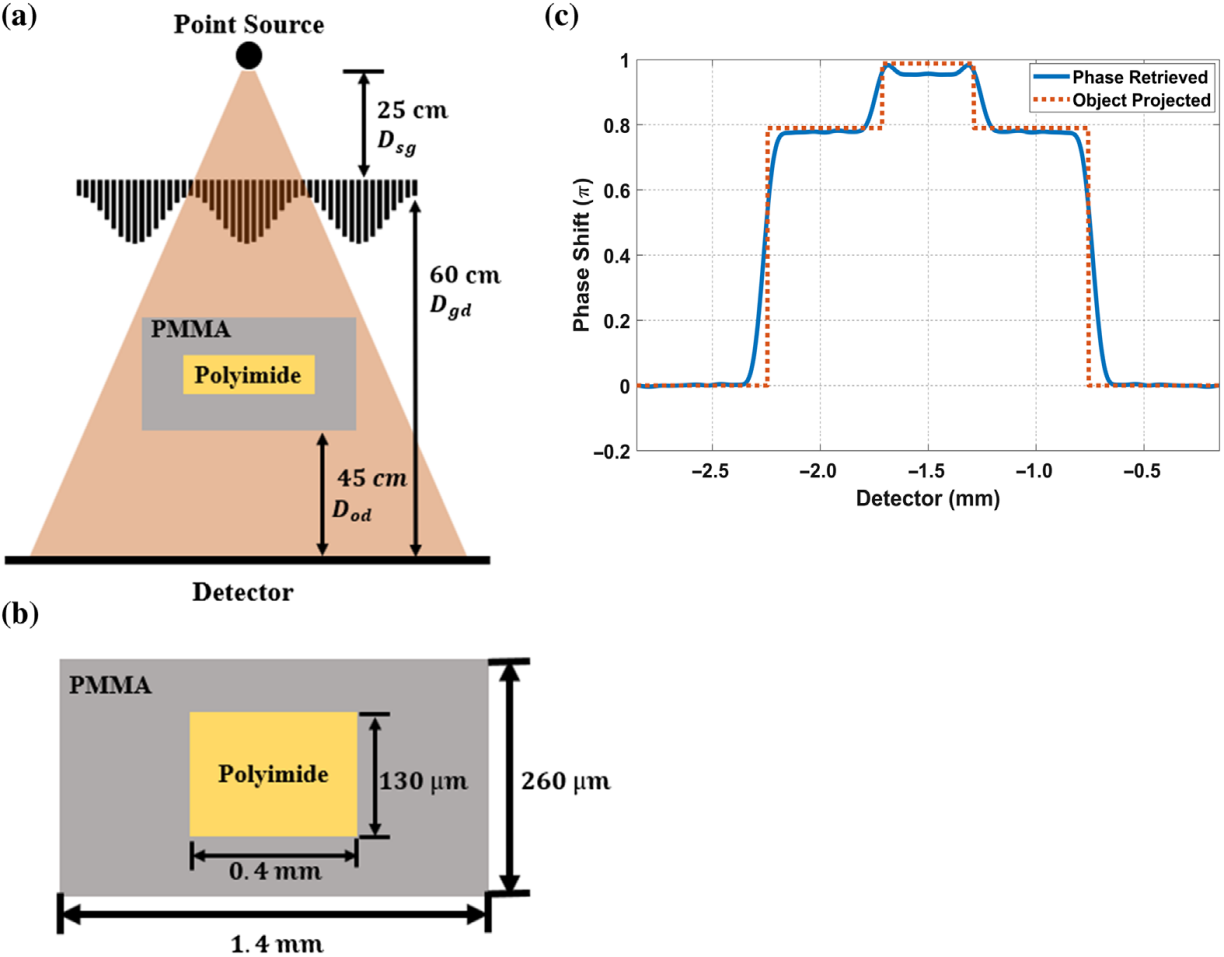
Phase profile retrieved from a simulated object projection from a point source at 40 keV. (a) The geometry of BCT and object projection. The source is 25 cm from MPG and the MPG-to-detector distance is 60 cm. The object is placed at 45 cm above the detector. (b) The simulated object is a polyimide slab in 0.4 mm width and 130  μm height embedded in a PMMA block in 1.4 mm width and 260  μm height. (c) The retrieved phase profile from fringes and the phase projection from the simulated object on the detector.

We also simulated with an object in BCT geometry, as shown in Fig. 16(a). The source-to-MPG distance is 25 cm, the MPG-to-detector distance is 60 cm, the object-to-detector distance is 45 cm, and the scanning energy of a point source is 40 keV. The simulated object, as shown in Fig. 16(b), is a polymethyl methacrylate or acrylic (PMMA) block of 1.4-mm width, 260-μm thickness, and corresponding 0.79π phase shift in the beam propagation direction at 40 keV. A polyimide block in 0.4-mm width, 130-μm thickness, and corresponding 1.6π phase shift is embedded at the center of the PMMA block. The projected phase profile of this object on the detector is retrieved by the single-shot method^25^ from fringes with phase sensitivity $\frac{D_{\mathrm{od}}}{W^{\prime }}=\frac{45\;\mathrm{cm}}{150\;\mu m}=3\times 10^{3}$. Figure 16(c) shows the projected phase profile of an object on the detector and the retrieved phase profile from fringes.

## 4 Discussion and Future Work

We show that a spatial MPG can be built to generate and control fringes that are visible on a standard detector (for example, flat panel with 50-μm resolution) without the absorption grating (analyzer) in between the phase grating and detector. The period of visible fringes can be controlled by the modulation period W parameter of the grating and system magnification. The visibility at a specific geometry (given $D_{sg}$ and $D_{gd}$) can be optimized by the height difference $\left(h_{2}-h_{1}\right)$ of the phase shifts of the low- and high levels of the grating.

Our point source simulation for a common BCT geometry shows only a small loss of visibility from 30.5% (monochromatic) to 27% (polychromatic) visibility. Similarly, using a line-source such as required for spatial coherence, the fringe visibility lowered from 30.5% to 28%.

The W is nominally chosen here to be ∼40 to 50  μm, which yields a fringe period of ∼150 to 300  μm at the detector (via magnification), which can be resolved with a 50-μm detector (such as used in the TLXI system^26^). But, in fact, there are other higher resolution CCD detectors as low as ∼25  μm can be potentially used for BCT.^16^^,^^27^ Note that, provided the period is resolvable with a small enough detector resolution, a smaller $W^{\prime }$ (i.e., W for the same geometry) is preferred. A smaller period yields higher visibility and higher phase sensitivity and provides better resolution of phase-image recovery if the single-shot Fourier method^25^ is used. From a spatial coherence point of view as well, a smaller W is preferred. For example, for spatial coherence, $l_{c}\approx P$ is preferred. For example, with spatial coherence, $l_{c}\approx P$, and fixed $L_{s}=\frac{\lambda D_{sg}}{lc}=P_{0}{ϒ}_{0}$, lowering the $W^{\prime }$ (i.e., W for the same geometry) will lower the $P_{0}$. The pitch of the $G_{0}$ grating (which is proportional to $W^{\prime }$) thereby increases the open-ratio ${ϒ}_{0}$.

The main objective with this system is to deliver the same signal-to-noise ratio for the attenuation image for the BCT acquisition, so the MPG-BCT system does not degrade the tried-and-tested attenuation images of standard BCT systems. In addition to similar quality attenuation images, the MPG BCT will provide phase and scatter images. Not having the x-ray absorbing analyzer, a similar dose to the patient fluence is improved by a factor of approximately two times with respect to clinical TLXI.^26^

Since the MPG is nearly transparent to x-rays we iterate that the attenuation image sensitivity will be preserved with respect to the BCT. Not having the analyzer further reduces the system cost compared with TLXI. The cost of making our grating is largely dependent on the pitch and size. A 0.6- to 1-μm pitch is adequate for BCT operating energies. These pitch values are well within a reasonable range of manufacturing cost for x-ray phase gratings.

It is important to note that the $G_{0}$ decouples the x-ray tube source from the fringe formation.^15^^,^^16^ The focal spot of the x-ray tube will not affect the fringes’ resolution directly if there is a source coherence grating $G_{0}$.^16^ As far as fringe formation is concerned, we have a series of small mutually incoherent “line-sources” determined by a $G_{0}$ open ratio that form registered fringes.^15^^,^^16^

However, it is also important to note that the focal spot blur of the x-ray tube source affects the object similarly as in noninterferometric x-ray imaging.^4^^–^^9^ With 1.5 to 2 magnification in our system and 0.3-mm source spot size, the effect of the focal spot on the detector is similar to other BCT systems.^4^^–^^9^

For our geometry, for focal spot size of nominally 0.3 mm, a 55-μm pinhole aperture will reduce the focal spot blur to under 50-μm pixel size. With similar current in mA as in conventional imaging, this will increase typical BCT imaging times from a few seconds to a few minutes. A breast holder may be used for imaging to reduce motion.^37^ Another option is a microfocus x-ray tube with about a 50-μm spot size, as has been used for BCT (e.g., University of Naples system^38^^–^^40^). Due to smaller allowable maximum currents, using microfocus x-ray will increase the imaging times, making a breast-holder essential.

If a lower resolution detector is used, the fringe period may need to be increased for adequate sampling. This can be achieved in a few ways. Keeping the distance from source to grating constant, increasing the distance from grating to detector would increase the fringe period via magnification (and visibility). However, typically, there are clinical limits of space in a hospital setting. Another option is to use an MPG with a higher W to obtain a larger fringe period in a clinically compatible source to detector distance.

The analyzer double functions as a scatter grid.^41^ However, in conventional BCT and mammography, the scatter grid not only absorbs fluence but also requires careful alignment; failing in this results in image artifacts leading to repeat scans.^42^ Hence, the current trend in BCT or mammogram is to perform grid-less scatter correction algorithms that produce equivalent image quality.^43^^–^^46^ In fact, for mammography, the scatter correction algorithm from Siemens, Inc., is now FDA approved and used in the clinic.^47^ Similar to the grid in the mammogram or CT, the analyzer absorbs fluence and careful alignment is an issue. Another consideration is that with the relatively large magnification due to the central placement of the object, BCT scatter effects are lower.^4^ In the future, we will estimate the effect of Compton scatter and build an iterative scatter correction algorithm for our system. This could be projection-based such as in mammography^44^^,^^45^ or in 3D object space as in BCT.^46^ Fast Monte-Carlo methods maybe used to expedite the algorithm, as is done for mammography or BCT.^45^^,^^46^^,^^48^

We are in communication with Microworks GmbH, Germany,^49^ to build preliminary MPG gratings suitable for breast imaging. In the future, we anticipate building a prototype by modifying existing TLXI systems in Pennington Biomedical Research Center, LSU, and at the synchrotron source at Louisiana State University Center for Advanced Microstructures and Devices.

## 5 Conclusion

We demonstrated a novel analyzer-less x-ray interferometer with a spatially sinusoidal MPG. We show 27% fringe visibility with a total detector-to-source distance at 95 cm, which is clinically realistic. Our system was able to deliver the attenuation image without dose or fluence detriment compared with conventional BCT while delivering phase and scatter images within the same acquisition.
